# Organosilicon Fluorescent Materials

**DOI:** 10.3390/polym15020332

**Published:** 2023-01-09

**Authors:** Zixu Chen, Shengyu Feng, Dengxu Wang

**Affiliations:** 1 National Engineering Research Center for Colloidal Materials & Key Laboratory of Special Functional Aggregated Materials, Ministry of Education, Shandong Key Laboratory of Advanced Organosilicon Materials and Technologies, School of Chemistry and Chemical Engineering, Shandong University, Jinan 250100, China; 2 College of Medical Engineering & the Key Laboratory for Medical Functional Nanomaterials, Jining Medical University, Jining 272067, China

**Keywords:** organosilicon materials, fluorescent materials, silane, polysiloxane, polyhedral oligomeric sesquisiloxane, chemosensor, bioimaging, organic light-emitting diodes

## Abstract

In the past few decades, organosilicon fluorescent materials have attracted great attention in the field of fluorescent materials not only due to their abundant and flexible structures, but also because of their intriguing fluorescence properties, distinct from silicon-free fluorescent materials. Considering their unique properties, they have found broad application prospects in the fields of chemosensor, bioimaging, light-emitting diodes, etc. However, a comprehensive review focusing on this field, from the perspective of their catalogs and applications, is still absent. In this review, organosilicon fluorescent materials are classified into two main types, organosilicon small molecules and polymers. The former includes fluorescent aryl silanes and siloxanes, and the latter are mainly fluorescent polysiloxanes. Their synthesis and applications are summarized. In particular, the function of silicon atoms in fluorescent materials is introduced. Finally, the development trend of organosilicon fluorescent materials is prospected.

## 1. Introduction

As an essential element of group IV in the periodic table, silicon ranks the second most abundant element in the earth’s crust (25.7% mass fraction) [1]. Compared to the element carbon, which also belongs to the group IV, silicon possesses a unique atom structure and electron distribution, including longer bond length, smaller steric hindrance, larger nuclear charge, and lower electronegativity [2,3]. More importantly, the silicon atom has five empty 3d orbitals and can accept electrons from adjacent heteroatoms or groups, forming a dπ-pπ conjugate or Si-X coordination bond (X represents heteroatoms, such as N, S, P). These unique features endow organosilicon materials with special optical properties [4,5,6], and intriguing performances, including flexibility, electrical insulation, UV resistance, good breathability, hydrophobicity, nontoxicity, and biocompatibility [7,8]. Therefore, the “organosilicon” concept has drawn intense attention since it appears, together with in-depth exploration and wide applications in common industries such as aerospace, medical equipment, electronics, construction, and energy [9,10,11,12].

Accompanied by the prosperity of new technologies and the fusion of subjects, exploring organosilicon materials with novel functions, such as fluorescence [6,13,14,15], catalysis [16], bioactivity [17], self-healing [18], etc., is of significance from the perspective of future development. Among numerous cutting-edge research areas, fluorescence-based applications, such as bioimaging, chemosensors, and organic light-emitting diodes (OLEDs), have attracted specific attention. The incorporation of organosilicon units into fluorescent materials could bring enhancement of materials’ properties and meet the requirement of those applications, such as photostability, chemical stability, biocompatibility, and higher fluorescence quantum yield. As a consequence, the concept of organosilicon fluorescent materials combining the characteristics of silicone and fluorescent units was put forward and in prosperity.

In the past decade, researchers concentrated on the development of organosilicon fluorescent materials and presented some reviews. For example, in 2015, Sun et al. reviewed arylsilanes and siloxanes as optoelectronic materials for organic light-emitting diodes [19]. In 2016, Ren et al. summarized the general synthetic methods and optoelectronic applications of polysiloxanes [8]. In 2021, Zuo et al. reviewed the unconventional fluorescent silicon-containing materials and Si-O-Si linked organosilicon materials applied in the area of bioimaging [3]. However, no comprehensive review was reported from the perspective of organosilicon fluorescent materials and their applications. In this review, the organosilicon fluorescent materials will be systematically classified and discussed. Specifically, their applications will be summarized in detail with a focus on chemosensors, bioimaging, and OLEDs.

## 2. Classification of Organosilicon Fluorescent Materials

### 2.1. Organosilicon Fluorescent Small Molecules

#### 2.1.1. Aryl Silanes

The first known and most widely used organosilicon luminescent compounds are aryl silanes, in which the silicon atom was directly connected with aryl groups. There are mainly two types of aryl silanes, including polyphenylsilanes and siloles.

Polyphenylsilanes, as the name implies, are composed of two or more phenyl groups connecting with the silicon atom or employing silicon atom as the central atom, and have high singlet and triplet state energy levels because the silicon atom blocks the conjugated structure between aryl units. Meanwhile, the tetrahedral conformation of polyphenylsilanes can effectively prevent intermolecular interactions in the solid state and form uniform and smooth amorphous films. As a result, polyphenylsilanes have been widely used as emitting materials, electron-transporting materials, or host materials in the field of OLEDs. In 2012, Andrienko et al. reported 2,8-bis(triphenylsilyl)dibenzofuran as the electron-transporting material in deep-blue OLEDs [20]. Due to the mesomeric effects, it significantly improved the electronic couplings by delocalizing the frontier orbitals and lowering the reorganization energy, resulting in high electron mobility and a small Poole−Frenkel slope. This work provided an effective method for designing host materials with high charge mobility.

**Figure 1:**
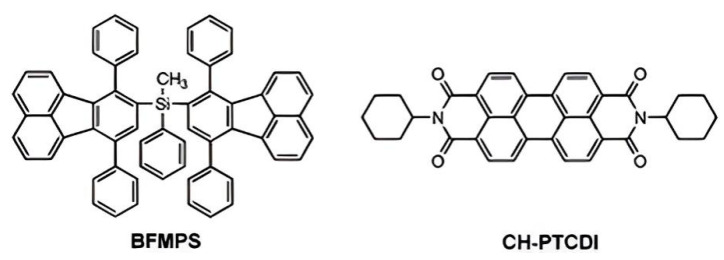
Molecular structures of BFMPS and CH-PTCDI [22]. Copyright 2013, RSC publishing.

Our group synthesized diphenylfluoranthene-functionalized silanes and found that they exhibited higher thermal stability and glass transition temperature than silicon-free diphenylfluoranthene, implying the introduction of silicon units can improve the thermal stability of aromatic compounds [21]. By mixing bis(7,10-diphenyl-fluoranthene) methylphenyl silane (BFMPS) and N, N-dicyclohexyl-perylene-3,4,9,10 tetracarboxylic acid bisimide (CH-PTCDI), a series of solutions and films with tunable emission colors were obtained (Figure 1) [22]. It is worth noting that a white-light emission with CIE coordinates (0.327, 0.339) can be easily achieved by tuning the ratio of BFMPS and CH-PTCDI. This intriguing finding suggests its potential as a good candidate in white optoelectronic devices.

**Figure 2:**
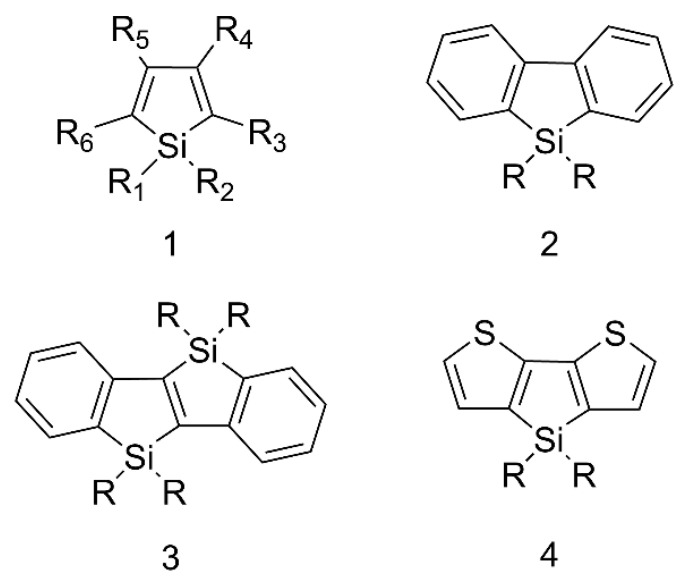
The representative types of siloles.

Siloles are a class of silicon-containing heterocyclic compounds with unique electronic structures and intriguing photophysical properties and the typical structures are shown in Figure 2. Siloles are considered as novel σ*-π* conjugated materials with a low LUMO energy level due to the interaction of the σ* orbital of the silicon-carbon bond with the π* orbital of butadiene [23]. By virtue of this unique electronic structure, siloles have high electron affinity and fast electron mobility, allowing them to be used as an electron transport layer to transport electrons in optoelectronic devices. More importantly, siloles are the first compounds discovered to have the aggregation-induced emission (AIE) effect, which Tang et al. proposed in 2001 [24,25]. They exhibit weak fluorescence in dilute solutions and enhanced fluorescence in the aggregated state, in contrast to conventional aggregation caused quenching (ACQ) [26]. Since the discovery of the AIE phenomenon in siloles as the cornerstone, there has been an explosion of AIE-related theories and material designs ranging from small molecules to polymers and clusters [27,28,29]. Fluorescent materials have reached new heights thanks to AIE materials. 

**Figure 3:**
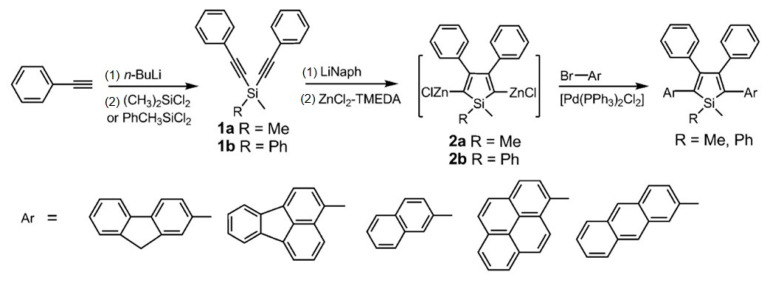
Synthetic routes to the siloles containing planar fluorescent chromophores. TMEDA = N,N,N′,N′-tetramethylethylenediamine [33]. Copyright 2014, Wiley publishing.

In 1969, Curtis et al. prepared the first 2,3,4,5-tetraphenysilole by reacting 1,2,3,4-tetraphenyl-1,4-dilithium dibutene with chlorosilane [30]. In 2001, Tang’s group discovered the AIE effect of silole [31]. Following that, Liu et al. concluded, based on theoretical calculations and an analysis of the crystal structure of 2,3,4,5-tetraphenylsilole, that the main cause of the AIE phenomenon was the blocked rotation of the 3,4-position benzene ring in the solid state, which suppressed the non-radiative energy consumption and resulted in the consumption of most of the singlet energy in the form of fluorescence [32]. In 2014, Chen et al. prepared a series of siloles, which were substituted in 2,5-positions with planar fluorescent chromophores (PFCs), including fluorene, fluoranthene, naphthalene, pyrene, and anthracene (Figure 3) [33]. These PFCs substituted siloles had higher emission efficiencies than PFCs in the aggregated state. Using these siloles as host emitters, highly efficient OLEDs were further fabricated with tunable emission colors from green (522 nm) to orange (582 nm). 

#### 2.1.2. Siloxanes

**Figure 4:**
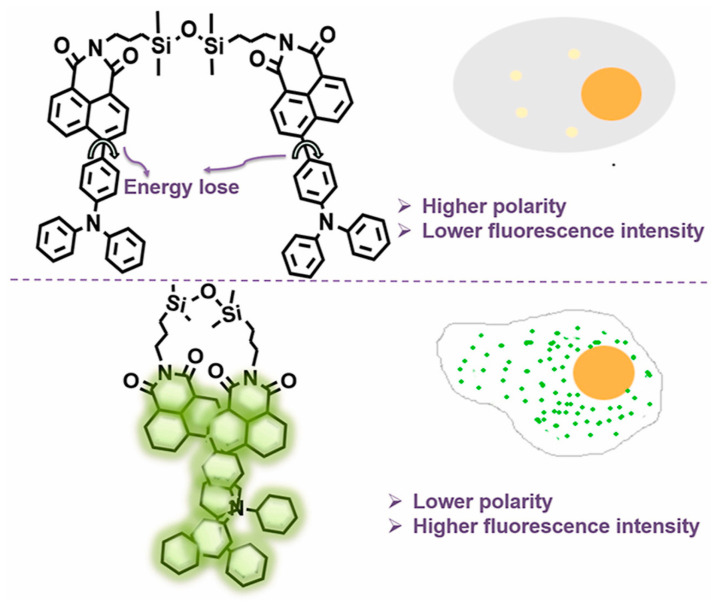
The structure of TDCQ [36]. Copyright 2021, Elsevier.

The versatility and widespread use of functionalized disiloxanes and silane coupling agents allows for the design and fabrication of a wide range of siloxane-based fluorescent molecules. A simple design strategy is modifying luminescent dyes onto siloxane. Lin et al. reported Si-O-Si bridged fluorescent molecules based on 1,3-bis(3-aminopropyl)tetramethyldisiloxane and aryl luminescent dyes [34,35,36,37]. As shown in Figure 4, the fluorescent siloxane-based probe (TDCQ) consisted a naphthalimide moiety accepting electron and a triphenylamine fragment providing electrons [36], which was used for monitoring polarity of lipid droplets (LDs) in cells. As the number of LDs differs between normal and cancer cells, the probe emitted stronger green fluorescence in cancer cells, providing an effective method for distinguishing between normal and cancer cells. Notably, it was reported that TDCQ possessed outstanding photostability and biocompatibility due to the uniqueness of the siloxane component. 

**Figure 5:**
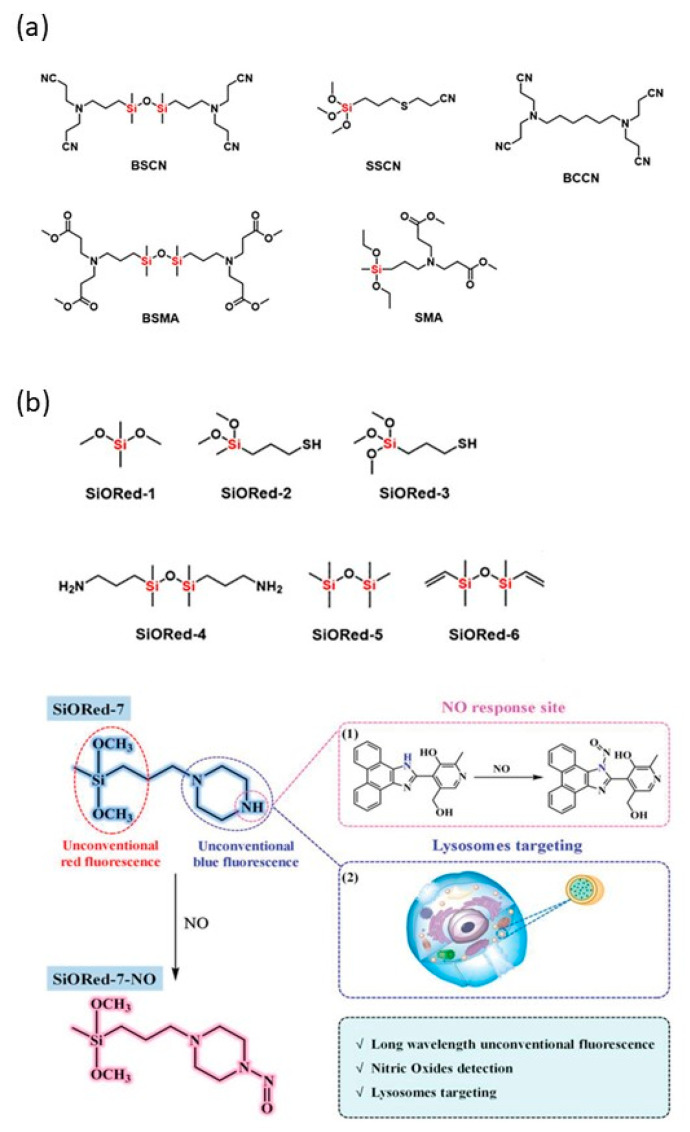
(**a**) Siloxane-based unconventional fluorescent compounds [40], Copyright 2017, Wiley publishing. (**b**) Structures of SiORed-1~SiORed-6 and the detection of NO by SiORed-7 [38], Copyright 2020, Wiley publishing.

Apart from conventional fluorescent molecules composing of aryl groups, in recent years, unconventional luminescent siloxane molecules that do not contain any of the conjugated systems have also received much attention and become an important branch in this field. In unconventional fluorescent molecules, electron-rich atoms such as sulfur and nitrogen are present and interact with silicon atom [38,39]. In addition, the flexible Si-O bond is conducive to fluorescent emission [40]. In 2017, we synthesized several unconventional siloxane-based compounds and the corresponding siloxane-free compounds via Michael addition or click chemistry (Figure 5a) [40]. The photophysical properties revealed that the siloxane-based compounds emitted ultraviolet light in dilute solutions with the maximum emission wavelength (λ_max_) at 425 nm, but no fluorescence was detected in the dilute solutions of siloxane-free compounds. Structural characterizations and density functional theory (DFT) calculations for siloxane-based compounds proved that nitrogen or sulfur atoms in siloxanes were able to coordinate with silicon atoms, leading to enhanced fluorescence emission. 

Most unconventional luminescent siloxane molecules have a low emission wavelength, which is mainly located in UV, blue and green regions. Zuo et al. [38] found Si–O bridged organoalkoxysilanes were capable of emitting unexpected red fluorescence (SiORed-1–6). Inspired by the desired red emission, SiO-Red-7 was further constructed and applied to detect NO in lysosomes and in vivo accompanied by a blue fluorescence quenching and a red fluorescence enhancement (Figure 5b). 

**Figure 6:**
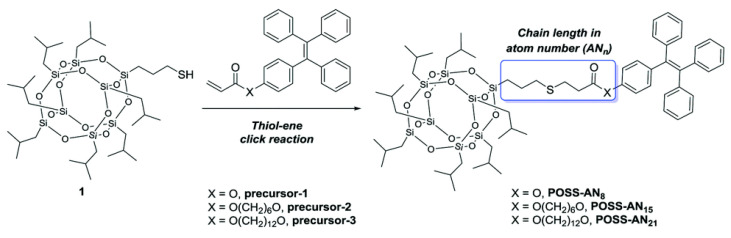
Synthesis of POSS-AN_8_, POSS-AN_15_ and POSS-AN_21_ [46]. Copyright 2016, Royal Society of Chemistry.

Polyhedral oligomeric silsesquioxanes (POSS) as a kind of siloxanes with unique cage structure have attracted intense attention in the last decade. POSS possess ideal 3D inorganic–organic hybrid structures, which consist of a rigid inorganic siloxane core and surrounding reactive organic functional groups (R groups). The R groups are diversified and adjustable [41]. It was worth noting that POSS units could suppress the self-quenching of organic luminescent dyes and further improve their quantum efficiency by virtue of their 3D scaffold structures [42]. Benefiting from this special feature, POSS have been considered an ideal platform for constructing fluorescent molecules [43,44,45]. As illustrated in Figure 6, Zhou et al. constructed three kinds of POSS-based fluorescent materials (POSS-AN_8_, POSS-AN_15_, POSS-AN_21_) by modifying different tetraphenylenes (TPE) onto a single-angle of POSS via thiol-ene click reaction [46]. These mono-TPE modified POSS molecules with AIE effect exhibited stronger fluorescence emission intensity than the TPE monomer, and could act as a sensitive fluorescence probe to monitor the self-assembly of POSS molecules in solvents.

Chanmungkalakul et al. prepared POSS-based fluorescent molecule bearing anthracene arms (AnSQ) by Heck reaction [47], and found that different spatial configurations of anthracene led to different fluorescent properties. As POSS has a 3D cubic structure, it provides a good platform to study the spatial configuration of anthracene. Moreover, AnSQ displayed solvent-dependent fluorescence and ions distinction performance, and thus can serve as a probe to detect or recognize different ions and solvents.

### 2.2. Polysiloxanes

Polysiloxanes consist of repeating Si-O-Si units as the main chain and organic groups attaching to the silicon atoms. Fluorescent polysiloxanes were commonly obtained by introducing fluorescent groups into polysiloxanes via suitable methods. Combining the advantages of polysiloxanes, such as good flexibility, excellent film-forming ability, high gas permeability, and good biocompatibility [48,49], polysiloxane-based fluorescent materials have been rapidly developed and found broad application prospects. On the basis of structure difference, three types of polysiloxane-based fluorescent materials, that is, linear, hyperbranched and cross-linked polysiloxanes, are summarized.

#### 2.2.1. Linear Polysiloxanes

Linear polysiloxane-based fluorescent materials are one of the most extensively researched fluorescent polysiloxanes, and are usually prepared by introducing fluorescent groups into the side chains or end groups of polysiloxanes. 

**Figure 7:**
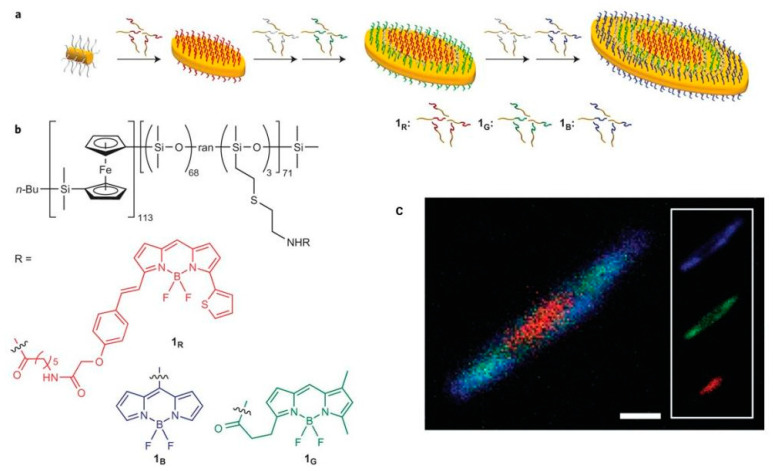
(**a**) Schematic diagram of block co-micelles. (**b**) Chemical structures of functional polysiloxanes. (**c**) LSCM images of these block co-micelles [50]. Copyright 2014, Nature Publishing Group.

Hudson et al. constructed controllable nanostructured polysiloxane in two dimensions by modifying three kinds of fluoroboron dipyrrole (BODIPY)-based dyes emitting red, green, and blue light, respectively, onto side chains of polysiloxane [50]. As shown in Figure 7, platelet hierarchical micelles with colors of red, green, and blue could be observed under laser scanning confocal microscopy (LSCM). The precise control of micelle was derived from crystallization-driven self-assembly of copolymer, and the multishaped hierarchical micelles, such as cylinder, single- and double-headed spear, were fabricated by this universal method. In the same year, this group prepared color-tunable fluorescent multiblock micelles by controlling the ratio of red–green–blue ternary colors on poly(ferrocenyldimethylsilane) [51], and successfully constructed white light-emitting nanomaterials with a CIE coordinate of (0.33, 0.33). In addition, clear fluorescent imaging multiblock micelles were obtained by the addition of nonfluorescent spacer into fluorescent polysiloxane, thereby revealing that this kind of polysiloxane has potential applications in bio-diagnostics, microdisplay technology, and encoded nanomaterials.

In 2019, Liang et al. introduced the fluorescent groups in the side chain of polysiloxane by Diels–Alder reaction between 7, 9-diphenyl-8H-cyclopenta acenaphthylen-8-one (DCPA) and vinyl-modified polysiloxane [52]. Attributed by the flexible Si-O-Si chains and π–π interactions of the fluorescent groups, this fluorescent polymer exhibited a porous spherical structure, resulting in a larger contact area with the analytes and a high detection efficiency. 

Zuo et al. synthesized fluorophore-terminated polysiloxanes (P1) by copolymerizing 1,3-bis(3-aminopropyl)tetramethyldisiloxane and terephthalaldehyde as well as using rhodamine B as the capping agent. P1 can selectively penetrate into apoptosis cells and exhibited sensitive response to Fe^3+^ ion [53]. Upon the addition of Fe^3+^ ion, the fluorescence emission color of P1 changed from blue to red. This phenomenon could be explained as follows. The unconventional blue fluorescence was caused by the silicon-nitrogen coordination. After adding Fe^3+^ ion, it disrupted this coordination, thus leading to the blue fluorescence quenching. Meanwhile, Fe^3+^ ion also attacked spirocyclic rhodamine-B ring, resulting in a “turn-on” red fluorescence.

#### 2.2.2. Hyperbranched Polysiloxanes

**Figure 8:**
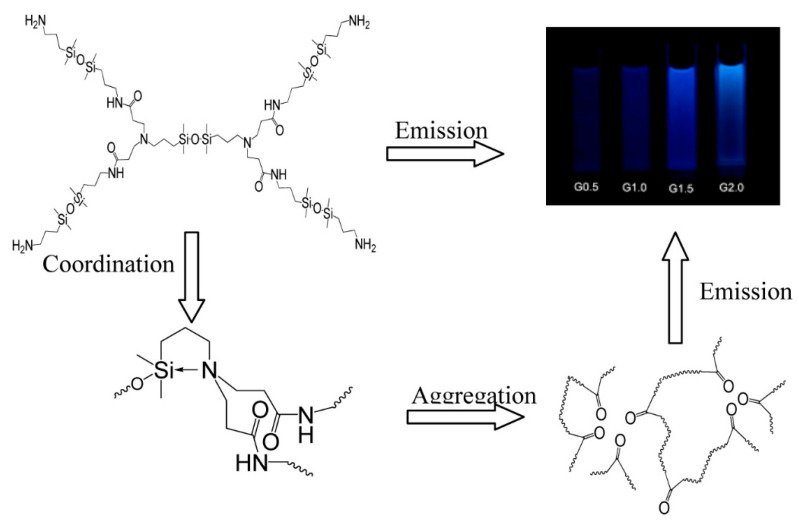
Scheme of the luminous mechanism of Si-PAMAM [5]. Copyright 2015, American Chemical Society.

Fluorescent hyperbranched polysiloxanes as an emerging class of fluorescent materials commonly exhibit unconventional fluorescence and the fluorophores within the structures generally including hydroxyl group [54], carbon–carbon double bond [55], epoxy group [56], amino group [57], carbonyl group [58] and heteroatoms [5]. Various unconventional fluorescence hyperbranched polysiloxanes have been developed prosperously due to their intriguing characteristics, such as low toxicity and good biocompatibility. A representative example is the siloxane−poly (amidoamine) (Si-PAMAM) dendrimers reported by our group in 2015 [5]. We found that Si-PAMAM unexpectedly emitted blue fluorescence, whereas the polyamide-amine dendrimer without silicon had almost no fluorescence. Experiment results and XPS characterization revealed that the aggregation of carbonyl groups caused Si-PAMAM to emit blue fluorescence, while N → Si coordination was conducive to this aggregation and improved the fluorescence intensity (Figure 8). 

In 2019, Feng et al. synthesized a fluorescent hyperbranched polysiloxane with carbonyl and vinyl groups (P1) [58]. Surprisingly, it exhibited unconventional fluorescence with high quantum yield of up to 43.9%. This finding can be explained by the fact that the silicon–oxygen bonds, carbonyl and vinyl groups in P1 interacted with each other to form “through-space” conjugation, resulting in an average electron density and a more stable molecular conformation.

**Figure 9:**
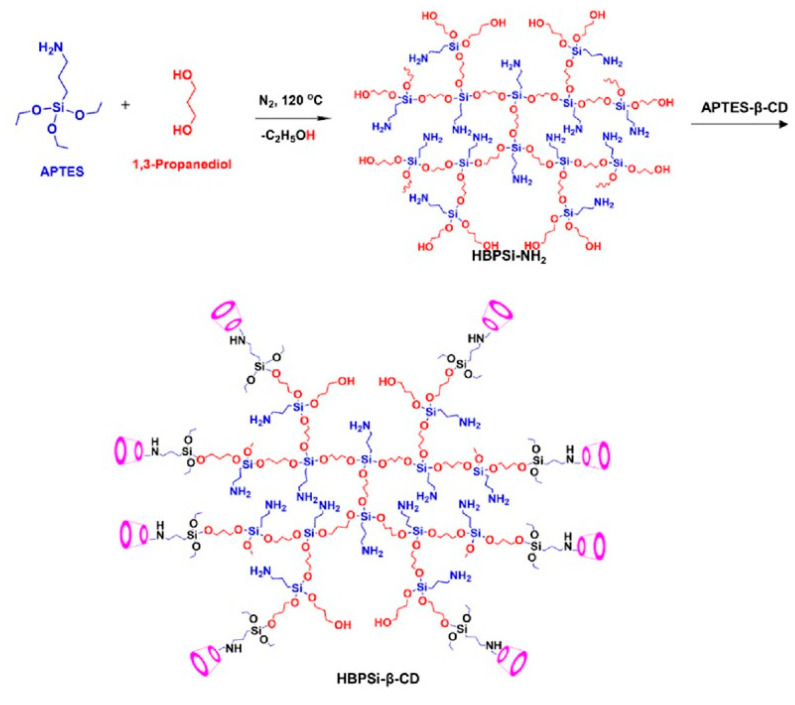
Synthesis route of HBPSi-β-CD [59]. Copyright 2022, American Chemical Society.

Very recently, β-cyclodextrin modified hyperbranched polysiloxane (HBPSi-β-CD) was fabricated and the synthetic route was illustrated as Figure 9 [59]. A multicolor emission ranging from blue to red with high quantum yields of 19.36%, 31.46%, 46.14%, and 44.84% was obtained when excited by 360, 420, 450, and 550 nm. The DFT calculations revealed that the various emissions were generated from the formed electron delocalization among the hydroxyl, amine, ether, and –Si(O)_3_ groups. In comparison to HBPSi-NH_2_, the introduction of β-cyclodextrin was beneficial to enhance the quantum yield and producing the multiple color emission of the hyperbranched polysiloxane. Notably, HBPSi-β-CD showed excellent performance in the detection of metal ions and visualization of controlled drug release.

#### 2.2.3. Cross-Linking Polysiloxanes

Polysiloxane-based fluorescent materials with cross-linked structures have been also developed rapidly. Since cross-linking polysiloxanes have the advantages of thermal endurance, aging resistance, and good flexibility, a number of fluorescent cross-linking polysiloxanes-based products with high performance could be manufactured by introducing fluorescent units into cross-linked polysiloxanes, such as fluorescent film materials [60], fluorescent elastomers [61,62,63], fluorescent flexible devices [64], etc. Polysiloxane as a good matrix material can provide abundant chemical sites for the attachment of fluorescent units. According to the interaction ways between fluorescent units and cross-linked polysiloxanes, they can be divided into physically doped and chemically bonded cross-linked polysiloxane-based fluorescent materials.

Physically doped materials are prepared by dispersing fluorescent materials into polysiloxanes, which are crosslinked by the additional cross-linking agents, while the fluorescent materials are physically mixed into the cross-linking system instead of participating chemical reaction. This preparation method is the earliest and most widely used strategy due to its simple operation and reliable effect for overcoming the aggregation quenching of luminescent groups. 

Buffa et al. prepared a red-emitting silicone rubber by doping the dye Lumogen Red 305 into the network and used it in luminescent solar concentrators [65]. Sato et al. doped four kinds of silicon nanoparticles emitting blue, green, orange, and red fluorescence into polysiloxane elastomers, providing a general method for the preparation of flexible transparent polymer-based composites [66]. In 2020, Hu et al. simultaneously added tricolor quantum dots (B-CDQs, Y-CulnS_2_@Zn, and Y-CulnS_2_@ZnS) into an aqueous solution containing 1-(3-(trimethoxysilyl)propyl)urea, and produced fluorescent polysiloxane materials (PSi) by the hydrolysis and polymerization reactions [67]. This method effectively overcame the aggregation-induced fluorescence quenching of quantum dots. The PSi emitted white fluorescence with the luminous efficiency as high as 127.5 lm W^−1^ and a CIE coordinate of (0.37, 0.37). 

**Figure 10:**
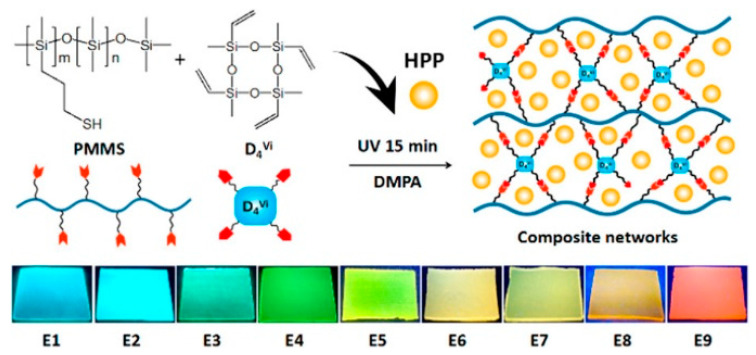
Preparation process of polysiloxane-based fluorescent composites (top). Photographs of the composites doped with hybrid porous polymers (bottom) [68]. Copyright 2018, American Chemical Society.

Our group [68] prepared the multicolored polysiloxane elastomers by physically blending fluorescent hybrid porous polymers (HPPs) into a polysiloxane matrix, following an efficient thiol−ene cross-linking reaction of poly[(mercaptopropyl)methylsiloxane] (PMMS) and tetramethyltetravinylcyclotetrasiloxane (D_4_^vi^). Thereinto, the HPPs were fabricated by the Heck reaction of octavinylsilsesquioxane and 4,4′-dibrombiphenyl and/or 1,3,6,8-tetrabromopyrene, and their colors could be easily regulated from blue to red by controlling the molar ratio of biphenyl and pyrene units (Figure 10). The HPPs were insoluble in common solvents; however, they could be well dispersed in the polysiloxane matrix when the amount of HPPs was below 20 mg per gram of PMMS. This work provided an effective approach to dope insoluble fluorophores into the polymer matrix and further develop fluorescent devices.

**Figure 11:**
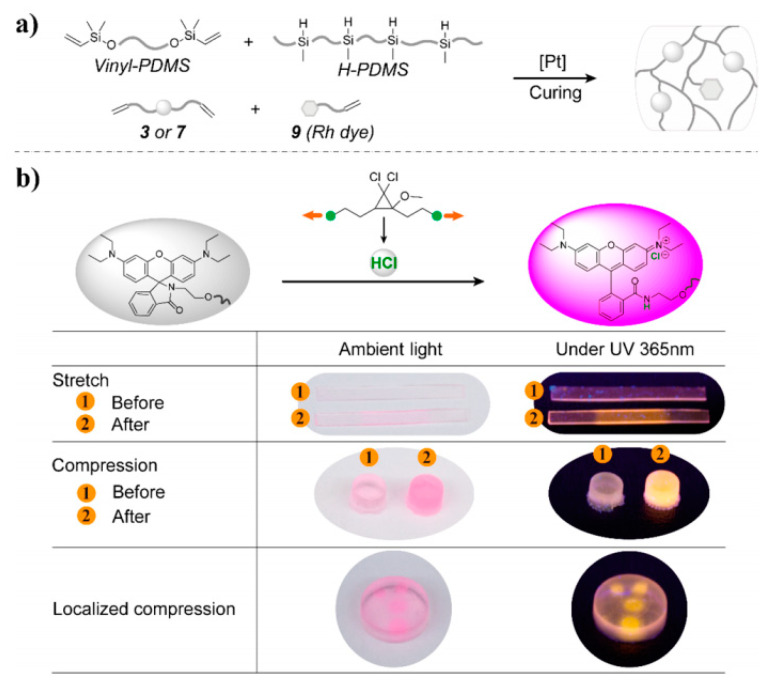
(**a**) Preparation of a PDMS elastomer, and (**b**) scheme of the luminous mechanism [69]. Copyright 2020, American Chemical Society.

Although the physical method for preparing cross-linking polysiloxanes is simple and convenient, the doping amount is limited due to the poor compatibility between fluorescent materials and polysiloxane substrates. In addition, the fluorescent materials may aggregate or precipitate within the crosslinked network after prolonged use, which affects the luminescence homogeneity and optical stability of the materials. To overcome this defect, researchers have introduced fluorescent units into cross-linked polysiloxane systems by chemical bonding. Lin et al. prepared stress-responsive polysiloxanes by reacting mechanoacid diene and rhodamine dye derivatives with poly(dimethylsiloxane) (PDMS) (Figure 11a) [69]. As shown in Figure 11b, when the material was stressed, the mechanical acid released protons to protonate the rhodamine dye, which caused the material to turn red under daylight and emit bright yellow light under 365 nm UV irradiation. 

Very recently, our group developed luminescent silicone elastomers via Schiff base reaction between NH_2_-PDMS-NH_2_ and aldehyde-modified tetraphenylene derivatives [62]. The elastomers showed excellent fluorescence properties, mechanical properties, thermal stability, self-healing, and recycle properties. 

The sol-gel method has been also widely utilized to fabricate cross-linking polysiloxanes due to its simple operation and low-temperature processing. Liu’s group selected silane pre-functionalized carbon dots (SiCDs) and silane coupling agents to synthesize a series of fluorescent gels (SiCD-Gel) by this method [70]. Notably, the loading amount of SiCDs in polysiloxane was adjustable from 0% to 100%. The properties of SiCD-Gel were comparable to or better than SiCDs in solutions.

## 3. Applications

### 3.1. Chemosensors

Attracted by the abundant categories and intriguing properties of organosilicon materials, more and more fluorescent organosilicon derivatives have been developed and their applications are explored. A facile and easily handled application is fluorescent chemosensors, which can response to a targeted analyst with corresponding fluorescent signal.

**Figure 12:**
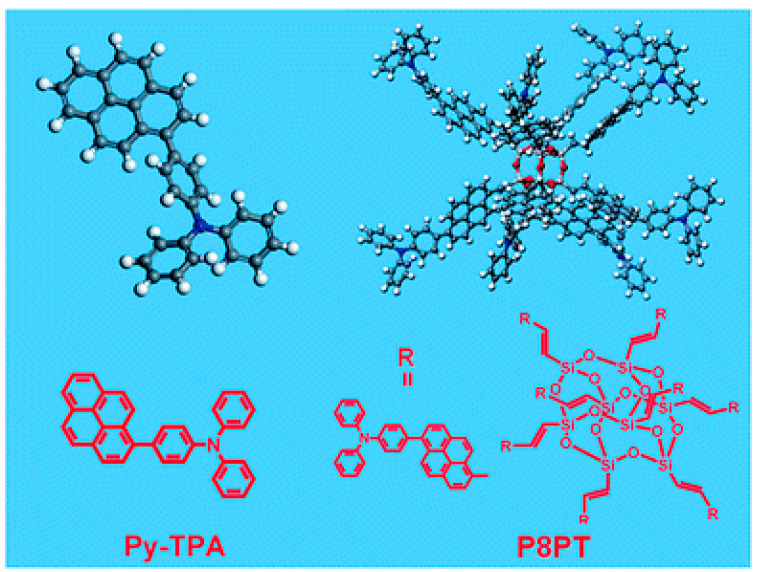
Structures of Py-TPA and P8PT [42]. Copyright 2015, Royal Society of Chemistry.

Detecting hazardous substance such as explosives is significant for human being and social stability, and researchers have devoted much attention to this field. Gao et al. designed eight triphenylaminopyrene-substituted POSS (P8PT) for the detection of nitrate ester explosives with higher molar extinction coefficient, specific surface area, and detection efficiency (92% upon exposure to saturated nitroglycerin vapor), compared to triphenylaminopyrene (Py-TPA) monomer [42]. As shown in Figure 12, theoretical simulations revealed that the energy level of P8PT matched well with nitrate, which was beneficial to improve the detection efficiency and selectivity.

High selectivity for detecting explosive is a sustaining concern for researchers. Current probes usually prefer to detect multinitro-substituted nitroaromatic compounds (NACs), typically 2,4,6-trinitrotoluene and 2,4,6-trinitrophenol [71,72,73,74]. A fluorescent probe for selectively detecting 4-nitrotoluene (NT) was developed by Gou et al. [75] and was synthesized by Heck reaction of 1-bromopyrene and vinyl-modified polysiloxanes. The research revealed that the selectivity was derived from the synergistic effect between steric hindrance and dipolar interaction. The flexible backbone structure of polysiloxane and formed rigid side-chain fluorophore provide an ideal platform for the highly selective detection of NT. 

Another meaningful application of chemosensor is detecting various ions, which are closely related to the environmental system [76,77,78]. A typical example is mercury ion, which as a heavy metal ion has greatly threatened the environment and human health. Our group developed a fluorescent POSS-based compound modified with imidazoline-2-selenone (POSS-Se) to simultaneously detect and remove Hg(II) ions [79]. POSS-Se could detect Hg(II) ions with two modes, including fluorescence “turn-on” phenomenon and color change from dark yellow to white observed by the “naked-eye”, and the limit of detection was very low to 8.48 ppb. Meanwhile, the removal amount of POSS-Se for Hg(II) ions was high up to 952 mg g^−1^.

Except for chemical analyst, the physical signals, such as force, temperature, and light, can also be effectively detected by organosilicon-based chemosenosrs [61,69,80,81,82]. Heras et al. reported carbazole-containing polysiloxane elastomers (LSCEs) to detect mechanical events by recording fluorescent signals [83]. To improve the detection performance, two conditions were investigated, i.e., the connection types of the carbazole fluorophores (end-on or side-on) to the elastomeric network and the length of the alkyl chain. The results showed that end-on carbazole fluorophores with short or medium flexibility were conducive to construct force sensors with higher efficiency. 

**Figure 13:**
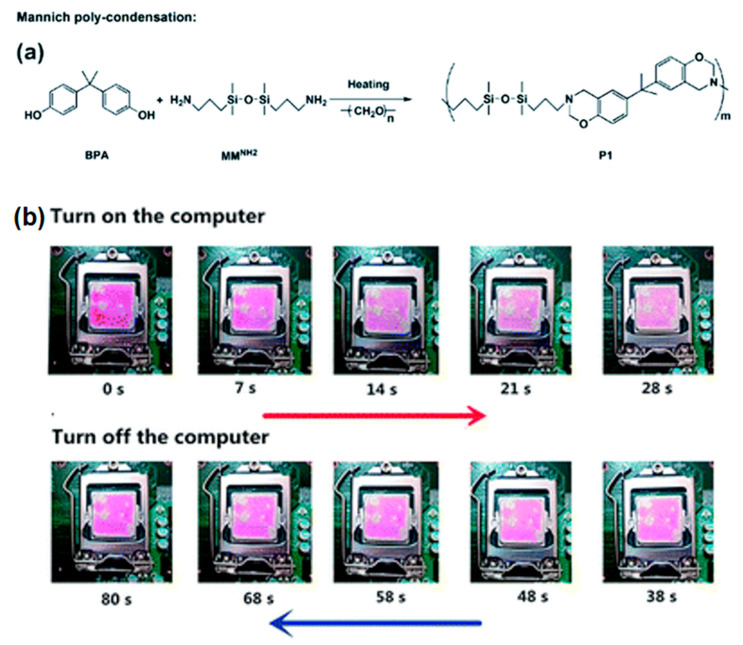
(**a**) Synthesis of P1 by Mannich polycondensation; and (**b**) digital images of a D1-non coated CPU with different running times illustrating the color changes [84]. Copyright 2018, Royal Society of Chemistry.

A thermally responsive polysiloxane (Dns-non) with unique two-photon luminescence was first reported by Zuo et al. [84]. The sensor was fabricated by incorporating rhodamine-B into benzoxazine-containing polysiloxanes (P1) (Figure 13). The fluorescence of Dns-non could switch from the “on” to “off” state reversibly along with the heating and cooling process. Furthermore, Dns-non was developed to a CPU temperature sensor by easily coating it onto the CPU surface and was utilized for monitoring the temperature of the CPU in situ by observing the color change. 

In conclusion, the introduction of organosilicon unit into chemosensors brings lots of new properties and improvements, such as higher contact surface area to analytes, special configuration and energy level, leading to a lower detection limit, higher fluorescence quantum yield and selectivity. 

### 3.2. Fluorescence Imaging

Fluorescence imaging is one of the most powerful methods for the spatial and temporal visualization of bio-species in living systems [3]. As organosilicon fluorescent materials possess good biocompatibility and low toxicity, they have promising application potential in bioimaging [53,85,86,87]. 

Two-photon fluorescence (TPF) bioimaging is attractive for its advantages over traditional one-photon fluorescence bioimaging, including increased penetration depth, good spatial resolution, negligible background fluorescence, etc. [88,89,90]. Chen et al. developed a two-photon fluorescent silole, i.e., 2,5-bis[5-(dimesitylboranyl)thiophen- 2-yl]-1-methyl-1,3,4-triphenylsilole ((MesB)_2_DTTPS), exhibiting a fluorescence emission peak at 598 nm and a high fluorescence quantum yield of 32% [91]. (MesB)_2_DTTPS was further encapsulated in lipid-PEG matrix to fabricate fluorescent organic dots, which were successfully applied to TPF imaging of MCF-7 cells and vivo visualization of the blood vascular of mouse muscle. In addition, (MesB)_2_DTTPS with aggregation-enhanced emission (AEE) could enhance the fluorescence efficiency of nanoaggregates.

**Figure 14:**
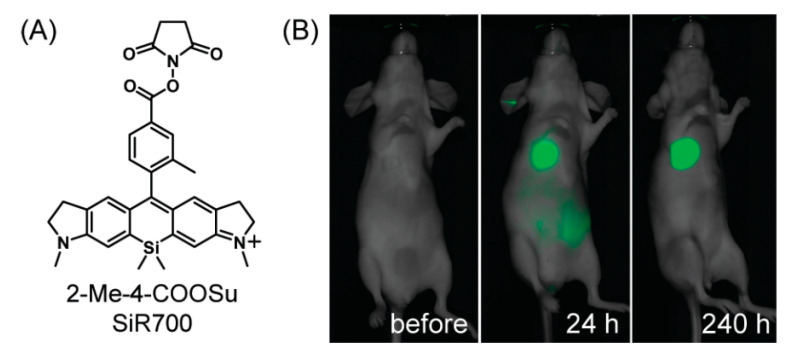
(**A**) The structure of 2-Me-4-COOSu SiR700. (**B**) In vivo tumor imaging with SiR700-labeled anti-tenascin-C antibody [94]. Copyright 2012, American Chemical Society.

The near-infrared (NIR) fluorescence imaging is also an intriguing method for biological applications because it possesses less light scattering and minimal photo-damage to biological samples [92,93]. Koide et al. prepared a novel near-infrared (NIR) wavelength-excitable fluorescent dye by modifying the Si−rhodamine scaffold [94]. As shown in Figure 14, the amine-reactive succinimidyl ester-bearing Si−rhodamine scaffold, 2-Me-4-COOSu SiR700, was fabricated for the labeling of biomolecules such as antibodies and proteins, and applied for vivo tumor imaging targeted to the extracellular matrix glycoprotein tanascin-C (TN-C). In 2019, a NIR-emitting silicon rhodamine-binding RNA aptamer (SiRA) was reported [95], and could respond to the environment together with the structure change of silicon rhodamines between the noncolored spirolactone and the fluorescent zwitterion. SiRA was used to visualize the expression of mRNA in live-cell imaging experiments, thereby providing a valuable example to the field of aptamer-based RNA imaging.

**Figure 15:**
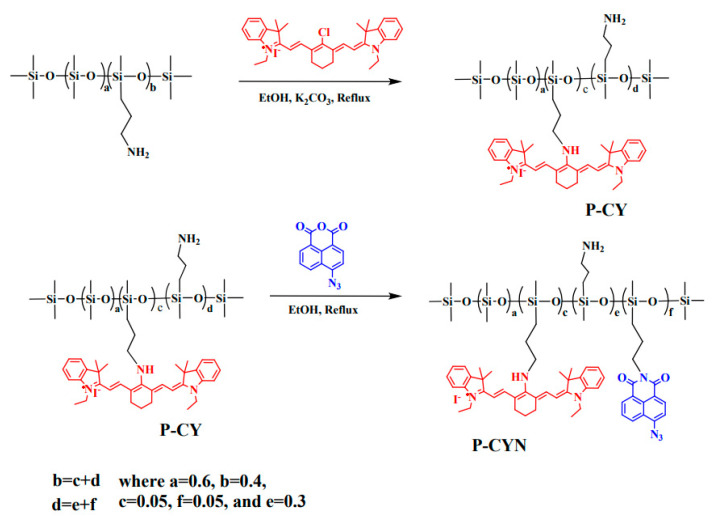
The preparation process of a fluorescent polysiloxane (P-CYN) with a dual fluorescent mode [96]. Copyright 2020, Royal Society of Chemistry.

Owing to the bright prospects of TPF and NIR in bioimaging, the development of new organosilicon materials with dual imaging modes draws researchers’ intense interest. In 2020, Zuo et al. prepared a fluorescent polysiloxane (P-CYN) with a dual fluorescent mode, TPF and NIR, for detecting NO and H_2_S in living organisms [96]. The preparation process is shown in Figure 15, where the heptamethine cyanine and heptamethine cyanine were step-wise modified on the side chain of polysiloxane. P-CYN displayed a near-infrared emission at 760 nm and the emission was quenched when it encountered NO. In contrast, when H_2_S was added to the P-CYN solution, the green fluorescence at 530 nm turned on, when excited by a wavelength of 405 nm or a two-photon excitation at 800 nm.

Notably, Zuo et al. found that organoalkoxysilanes emitted red unconventional fluorescence and were successfully applied to nitric oxide detection in cells, expanding the fluorescent material design strategy [38]. This work inspired researchers to deeply exploit the special optical properties of organosilicon materials. More applications based on organosilicon fluorescent imaging materials are expected to be explored, such as disease monitoring, drug delivery, and photodynamic therapy.

### 3.3. Organic Light-Emitting Diodes (OLEDs)

Owing to the growing demand for low energy consumption and high performance of display and lighting equipment, OLEDs have received much attention for applications in display devices and solid-state lighting and full-color flat-panel. The organosilicon units bring significant improvement to the physical and electronic properties of OLEDs and have been widely used as emitters, charge-transport and host materials in OLEDs [19].

**Figure 16:**
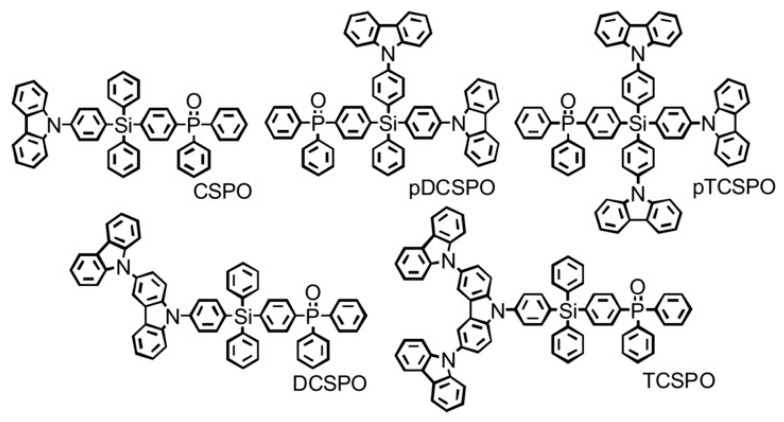
Chemical structures of five tetra-arylsilane based host materials [97]. Copyright 2012, Wiley-VCH.

Liu et al. designed a series of tetraarylsilane-based host materials of blue-phosphorescent devices with a high external quantum efficiency (EQE), where tetraphenylsilane as the core was functionalized with different ratios of *p*-type carbazole and *n*-type phosphine oxide units (Figure 16), leading to a charge-injecting and transporting balance [97]. Among them, 4-diphenylphosphine oxide-4′-[3-(9H-carbazole-9-yl)-9H-carbazol-9-yl]-tetraphenylsilane (DCSPO) achieved the highest current efficiency of 49.4 cd A^−1^ and an external quantum efficiency of 27.5%.

Recently, Vasilopoulou et al. developed a high-efficiency blue OLED with a below-bandgap turn-on voltage of 2.5 V and a high EQE of 41.2% by taking diphenyl[4-(triphenylsilyl)phenyl] phosphine oxide (TSPO1) as the acceptor constituent [11].

**Figure 17:**
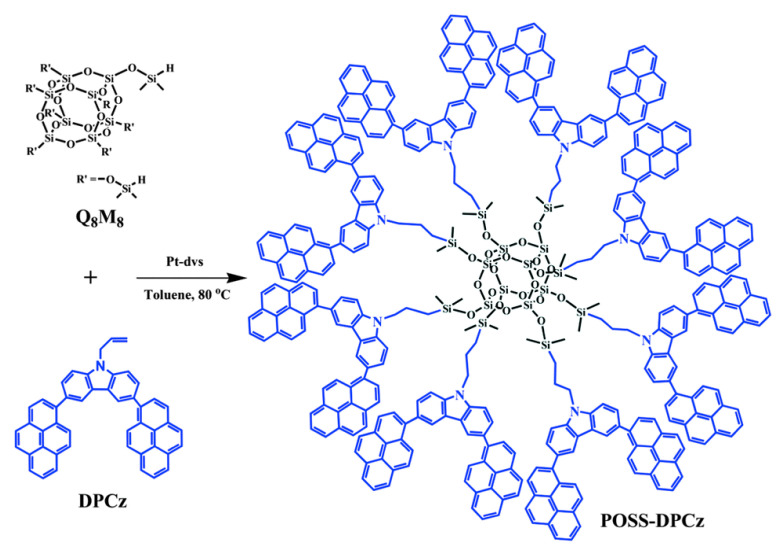
Synthetic procedure of POSS–DPCz [101]. Copyright 2016, Royal Society of Chemistry.

In recent years, POSS and their derivatives have drawn increasing attention in the fields of OLEDs because POSS units can enhance the thermal and mechanical properties as well as fluorescence quantum yields of OLEDs by taking advantages of the POSS units, including high thermal stability, rigid cubic geometry, and the three-dimensional structure [98,99,100]. Cheng et al. prepared 3,6-dipyrenylcarbazole-POSS hybrids (POSS-DPCz) by hydrosilylation reaction (Figure 17) [101]. The 3D POSS effectively suppressed the aggregation of conjugated fluorescent dyes (DPCz) and improved the fluorescence quantum yields of optical devices. The OLEDs made from POSS-DPCz emitted a stable blue light (450 nm) with a maximum brightness of 8900 cd/m^2^, which was almost two-fold higher than the DPCz-based OLEDs.

A POSS-armed poly(3,4-ethylenedioxyselenophene) (EDOS−POSS) was obtained by a two-step reaction of alkyl-substituted POSS and 3,4-ethylenedioxyselenophene (EDOS) [98]. Due to the presence of POSS on the polymer side chains, PEDOS-POSS exhibited good optical contrast (59%), coloring efficiency (95% at 593 cm^2^/C), and a low switching time (0.7 s). In addition, the PEDOS-POSS polymer films displayed excellent electrochemical stability and maintained 76% electroactivity after 5000 cycles.

**Figure 18:**
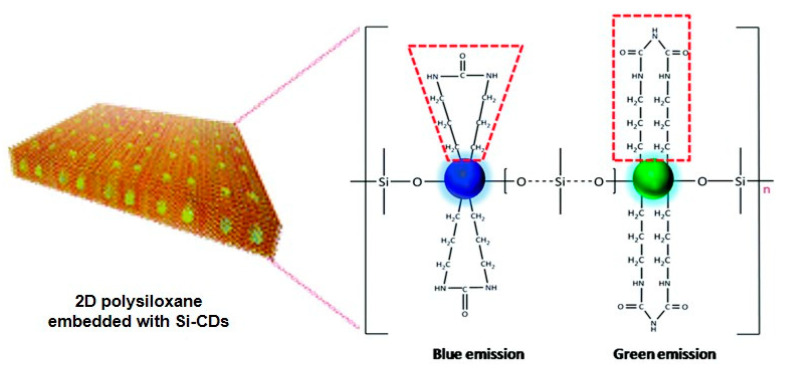
Schematic diagram of the 2D polysiloxane [102]. Copyright 2020, Royal Society of Chemistry.

Another example is the OLED constructed by coating fluorescent polysiloxane composites on the LED chips [102]. As shown in Figure 18, the hybrid composites were composed of polysiloxane and Si-doped carbon dots (P-E-Si-CDs), which exhibited outstanding photostability, high thermal stability, and a high photoluminescence quantum yield of 82.8%. The fabricated OLEDs based on UV and blue-emitting LED chips showed safe warm white light emission and adjustable white emission with a high color rendering index of up to 91, respectively. This work provided a feasible method for realizing high-performance carbon dots-doped LED lighting.

To sum up, due to the facile processability, structural versatility, excellent resistance to thermal, chemical and irradiation degradations, various organosilicon units including arylsilanes, siloxanes, POSS and polysiloxane have served as building blocks to construct OLEDs with high optoelectronic performance and stable structure.

## 4. Conclusions and Outlook

Organosilicon fluorescent materials have been flourishing in the last decade due to their excellent designability and properties. They have brought new functions and opened a new horizon in the area of fluorescent applications. On the basis of the achievements and new development tendency of these materials, we cover the emerging aspects of the recent progress in developing various organosilicon fluorescent materials from two basic categories, i.e., small molecules and polysiloxanes. Furthermore, their promising applications, such as chemosensors, bioimaging, and OLEDs, are collected and discussed to provide a reference for future research.

Although organosilicon fluorescent materials have wide-range applications and abundant species, there are still limitations that need to be solved. Firstly, it would be more relevant to investigate the roles that a silicon atom or siloxane unit plays in the fluorescence mechanism. For example, some theories have been put forward for the silicon-assisted unconventional fluorescence, e.g., “silicon induced aggregation luminescence”, “silicon-heteroatom coordination induced luminescence”, “Si-O bond and other chromophores form space conjugation and luminescence”, etc. However, there exist controversial arguments, and more experiments and theoretical calculations should be carried out to further investigate the mechanism. Secondly, more luminescence modes are expected to be exploited, such as long-persistent luminescence and up-converted luminescence. Moreover, it is a significant direction in designing more organosilicon fluorescent materials with higher fluorescent performance (i.e., quantum yield, color purity, photostability) by fully taking advantage of the unique electron contribution of silicon atoms. Current achievements are anticipated to generate more interest and attention from researchers and to further promote developments of organosilicon fluorescent materials. We also believe that the organosilicon fluorescent area will have a prosperous future in diverse research frontiers.

## Data Availability

Not applicable.

## References

[1] Liang J., Huang C., Gong X. (2019). Silicon Nanocrystals and Their Composites: Syntheses, Fluorescence Mechanisms, and Biological Applications. ACS Sustain. Chem. Eng..

[2] Baceiredo A., Kato T., Lee V.Y. (2017). Chapter 9: Multiple Bonds to Silicon (Recent Advances in the Chemistry of Silicon Containing Multiple Bonds). Organosilicon Compounds.

[3] Zuo Y., Liang X., Yin J., Gou Z., Lin W. (2021). Understanding the significant role of Si-O-Si bonds: Organosilicon materials as powerful platforms for bioimaging. Coord. Chem. Rev..

[4] Zuo Y., Gou Z., Quan W., Lin W. (2021). Silicon-assisted unconventional fluorescence from organosilicon materials. Coord. Chem. Rev..

[5] Lu H., Feng L., Li S., Zhang J., Lu H., Feng S. (2015). Unexpected Strong Blue Photoluminescence Produced from the Aggregation of Unconventional Chromophores in Novel Siloxane–Poly(amidoamine) Dendrimers. Macromolecules.

[6] Cao J., Zuo Y., Lu H., Yang Y., Feng S. (2018). An unconventional chromophore in water-soluble polysiloxanes synthesized via thiol-ene reaction for metal ion detection. J. Photochem. Photobiol. A Chem..

[7] Karaman D.Ş., Sarparanta M.P., Rosenholm J.M., Airaksinen A.J. (2018). Multimodality Imaging of Silica and Silicon Materials In Vivo. Adv. Mater..

[8] Ren Z., Yan S. (2016). Polysiloxanes for optoelectronic applications. Prog. Mater. Sci..

[9] Eduok U., Faye O., Szpunar J. (2017). Recent developments and applications of protective silicone coatings: A review of PDMS functional materials. Prog. Org. Coat..

[10] Vasilopoulou M., Yusoff A.R.B.M., Daboczi M., Conforto J., Gavim A.E.X., da Silva W.J., Macedo A.G., Soultati A., Pistolis G., Schneider F.K. (2021). High efficiency blue organic light-emitting diodes with below-bandgap electroluminescence. Nat. Commun..

[11] Yang J., Li W., Mu B., Xu H., Hou X., Yang Y. (2022). Simultaneous toughness and stiffness of 3D printed nano-reinforced polylactide matrix with complete stereo-complexation via hierarchical crystallinity and reactivity. Int. J. Biol. Macromol..

[12] Colpani A., Fiorentino A., Ceretti E. (2020). Feasibility analysis and characterization of an extrusion-based AM process for a two-component and biocompatible silicone. J. Manuf. Process..

[13] Dorneanu P.P., Homocianu M., Tigoianu I.R., Airinei A., Zaltariov M., Cazacu M. (2015). Solvent effects on the photophysical properties of poly[1,4-dihydroxyanthraquinoneimine-1,3-bis(phenylene-ester-methylene)tetramethyldisiloxane]. Spectrochim. Acta Part A Mol. Biomol. Spectrosc..

[14] Vlad A., Zaltariov M.-F., Shova S., Cazacu M., Avadanei M., Soroceanu A., Samoila P. (2016). New Zn(II) and Cu(II) complexes with in situ generated N_2_O_2_ siloxane Schiff base ligands. Polyhedron.

[15] Zaltariov M.-F., Cazacu M., Racles C., Musteata V., Vlad A., Airinei A. (2014). Metallopolymers based on a polyazomethine ligand containing rigid oxadiazole and flexible tetramethyldisiloxane units. J. Appl. Polym. Sci..

[16] Kung M.C., Riofski M.V., Missaghi M.N., Kung H.H. (2014). Organosilicon platforms: Bridging homogeneous, heterogeneous, and bioinspired catalysis. Chem. Commun..

[17] Loman-Cortes P., Binte Huq T., Vivero-Escoto J.L. (2021). Use of Polyhedral Oligomeric Silsesquioxane (POSS) in Drug Delivery, Photodynamic Therapy and Bioimaging. Molecules.

[18] Kathan M., Kovaříček P., Jurissek C., Senf A., Dallmann A., Thünemann A.F., Hecht S. (2016). Control of Imine Exchange Kinetics with Photoswitches to Modulate Self-Healing in Polysiloxane Networks by Light Illumination. Angew. Chem. Int. Ed..

[19] Sun D., Ren Z., Bryce M.R., Yan S. (2015). Arylsilanes and siloxanes as optoelectronic materials for organic light-emitting diodes (OLEDs). J. Mater. Chem. C.

[20] May F., Al-Helwi M., Baumeier B., Kowalsky W., Fuchs E., Lennartz C., Andrienko D. (2012). Design rules for charge-transport efficient host materials for phosphorescent organic light-emitting diodes. J. Am. Chem. Soc..

[21] Wang H., Liang Y., Wang Y., Xie H., Feng L., Lu H., Feng S. (2014). The strategy to improve thermal and optical properties of diphenylfluoranthene based on silicon-cored derivatives. RSC Adv..

[22] Wang H., Xie H., Liang Y., Feng L., Cheng X., Lu H., Feng S. (2013). Color-tunable organic composite nanoparticles based on perylene tetracarboxylic-diimides and a silicon-cored fluoranthene derivate. J. Mater. Chem. C.

[23] Zhao Z., He B., Tang B.Z. (2015). Aggregation-induced emission of siloles. Chem. Sci..

[24] Liu Y., Tang Y., Barashkov N.N., Irgibaeva I.S., Lam J.W.Y., Hu R., Birimzhanova D., Yu Y., Tang B.Z. (2010). Fluorescent Chemosensor for Detection and Quantitation of Carbon Dioxide Gas. J. Am. Chem. Soc..

[25] Ding D., Liang J., Shi H., Kwok R.T.K., Gao M., Feng G., Yuan Y., Tang B.Z., Liu B. (2014). Light-up bioprobe with aggregation-induced emission characteristics for real-time apoptosis imaging in target cancer cells. J. Mater. Chem. B.

[26] Huang M., Yu R., Xu K., Ye S., Kuang S., Zhu X., Wan Y. (2016). An arch-bridge-type fluorophore for bridging the gap between aggregation-caused quenching (ACQ) and aggregation-induced emission (AIE). Chem. Sci..

[27] Hu R., Qin A., Tang B.Z. (2020). AIE polymers: Synthesis and applications. Prog. Polym. Sci..

[28] Kong Y.-J., Yan Z.-P., Li S., Su H.-F., Li K., Zheng Y.-X., Zang S.-Q. (2020). Photoresponsive Propeller-like Chiral AIE Copper(I) Clusters. Angew. Chem. Int. Ed..

[29] Würthner F. (2020). Aggregation-Induced Emission (AIE): A Historical Perspective. Angew. Chem. Int. Ed..

[30] Curtis M.D. (1969). Synthesis and Reactions of Some Functionally Substituted Sila- and Germacyclopentadienes. J. Am. Chem. Soc..

[31] Luo J., Xie Z., Lam J.W.Y., Cheng L., Chen H., Qiu C., Kwok H.S., Zhan X., Liu Y., Zhu D. (2001). Aggregation-Induced Emission of 1-Methyl-1,2,3,4,5-Pentaphenylsilole. Chem. Commun..

[32] Yu G., Yin S., Liu Y., Chen J., Xu X., Sun X., Ma D., Zhan X., Peng Q., Shuai Z. (2005). Structures, electronic states, photoluminescence, and carrier transport properties of 1,1-disubstituted 2,3,4,5-tetraphenylsiloles. J. Am. Chem. Soc..

[33] Chen B., Jiang Y., He B., Zhou J., Sung H.H.Y., Williams I.D., Lu P., Kwok H.S., Qiu H., Zhao Z. (2014). Synthesis, structure, photoluminescence, and electroluminescence of siloles that contain planar fluorescent chromophores. Chem. Asian J..

[34] Zhang Y., Zuo Y., Yang T., Gou Z., Wang X., Lin W. (2019). Novel fluorescent probe with a bridged Si-O-Si bond for the reversible detection of hypochlorous acid and biothiol amino acids in live cells and zebrafish. Analyst.

[35] Wang X., Zuo Y., Zhang Y., Yang T., Lin W. (2020). An ICT-based fluorescent probe with bridging Si–O–Si bonds for visualizing hydrogen sulfide in lipid droplets and its application. Anal. Methods.

[36] Gai F., Zuo Y., Lin W. (2021). Detecting lipid droplets polarity: Silicone-based unique fluorescent probe for cancer diagnosis in living cells. Talanta.

[37] Zuo Y., Wang X., Lin W. (2021). Four-armed functional siloxane enables ratiometric unconventional fluorescence for the detection of ONOO^−^. Sens. Actuators B Chem..

[38] Zuo Y., Yang T., Gou Z., Tian M., Dong B., Lin W. (2020). Robust Organoalkoxysilanes as Red Unconventional Fluorescent Platform. Adv. Funct. Mater..

[39] Zuo Y., Wang D., Zhang J., Feng S. (2014). Multifunctional alkoxysilanes prepared by thiol–yne “click” chemistry: Their luminescence properties and modification on a silicon surface. RSC Adv..

[40] Lu H., Hu Z., Feng S. (2017). Nonconventional Luminescence Enhanced by Silicone-Induced Aggregation. Chem. Asian J..

[41] Zhou H., Ye Q., Xu J. (2017). Polyhedral oligomeric silsesquioxane-based hybrid materials and their applications. Mater. Chem. Front..

[42] Gao Y., Xu W., Zhu D., Chen L., Fu Y., He Q., Cao H., Cheng J. (2015). Highly efficient nitrate ester explosive vapor probe based on multiple triphenylaminopyrenyl-substituted POSS. J. Mater. Chem. A.

[43] Zuo Y., Wang X., Yang Y., Huang D., Yang F., Shen H., Wu D. (2016). Facile preparation of pH-responsive AIE-active POSS dendrimers for the detection of trivalent metal cations and acid gases. Polym. Chem..

[44] Cabrera-González J., Ferrer-Ugalde A., Bhattacharyya S., Chaari M., Teixidor F., Gierschner J., Núñez R. (2017). Fluorescent carborane–vinylstilbene functionalised octasilsesquioxanes: Synthesis, structural, thermal and photophysical properties. J. Mater. Chem. C.

[45] Sun M., Su Y., Yang W., Zhang L., Hu J., Lv Y. (2019). Organosiloxane and Polyhedral Oligomeric Silsesquioxanes Compounds as Chemiluminescent Molecular Probes for Direct Monitoring Hydroxyl Radicals. Anal. Chem..

[46] Zhou H., Li J., Chua M.H., Yan H., Ye Q., Song J., Lin T.T., Tang B.Z., Xu J. (2016). Tetraphenylethene (TPE) modified polyhedral oligomeric silsesquioxanes (POSS): Unadulterated monomer emission, aggregation-induced emission and nanostructural self-assembly modulated by the flexible spacer between POSS and TPE. Chem. Commun..

[47] Chanmungkalakul S., Ervithayasuporn V., Boonkitti P., Phuekphong A., Prigyai N., Kladsomboon S., Kiatkamjornwong S. (2018). Anion identification using silsesquioxane cages. Chem. Sci..

[48] Wolf M.P., Salieb-Beugelaar G.B., Hunziker P. (2018). PDMS with designer functionalities—Properties, modifications strategies, and applications. Prog. Polym. Sci..

[49] Abe Y., Gunji T. (2004). Oligo- and polysiloxanes. Prog. Polym. Sci..

[50] Hudson Z.M., Boott C.E., Robinson M.E., Rupar P.A., Winnik M.A., Manners I. (2014). Tailored hierarchical micelle architectures using living crystallization-driven self-assembly in two dimensions. Nat. Chem..

[51] Hudson Z.M., Lunn D.J., Winnik M.A., Manners I. (2014). Colour-tunable fluorescent multiblock micelles. Nat. Commun..

[52] Liang Y., Xu L., Qu F., Tang K., Wang H., Yu W.W. (2019). A silicone polymer modified by fluoranthene groups as a new approach for detecting nitroaromatic compounds. Polym. Chem..

[53] Zuo Y., Yang T., Wang X., Zhang Y., Tian M., Gou Z., Lin W. (2019). Visualizing the cell ferroptosis via a novel polysiloxane-based fluorescent schiff base. Sens. Actuators B Chem..

[54] Zhang Z., Gu A., Liang G., Yuan L., Zhuo D. (2013). A Novel Hyperbranched Polysiloxane Containing Epoxy and Phosphaphenanthrene Groups and its Multi-Functional Modification of Cyanate Ester Resin. Soft Mater..

[55] Niu S., Yan H., Chen Z., Li S., Xu P., Zhi X. (2016). Unanticipated bright blue fluorescence produced from novel hyperbranched polysiloxanes carrying unconjugated carbon–carbon double bonds and hydroxyl groups. Polym. Chem..

[56] Niu S., Yan H., Li S., Tang C., Chen Z., Zhi X., Xu P. (2016). A multifunctional silicon-containing hyperbranched epoxy: Controlled synthesis, toughening bismaleimide and fluorescent properties. J. Mater. Chem. C.

[57] Niu S., Yan H., Chen Z., Yuan L., Liu T., Liu C. (2016). Water-Soluble Blue Fluorescence-Emitting Hyperbranched Polysiloxanes Simultaneously Containing Hydroxyl and Primary Amine Groups. Macromol. Rapid Commun..

[58] Feng Y., Bai T., Yan H., Ding F., Bai L., Feng W. (2019). High Fluorescence Quantum Yield Based on the Through-Space Conjugation of Hyperbranched Polysiloxane. Macromolecules.

[59] Bai L., Yang P., Guo L., Liu S., Yan H. (2022). Truly Multicolor Emissive Hyperbranched Polysiloxane: Synthesis, Mechanism Study, and Visualization of Controlled Drug Release. Biomacromolecules.

[60] Zuo Y., Cao J., Feng S. (2015). Sunlight-Induced Cross-Linked Luminescent Films Based on Polysiloxanes andd-Limonene via Thiol-ene “Click” Chemistry. Adv. Funct. Mater..

[61] Wang N., Feng L., Xu X.-D., Feng S. (2022). Dynamic Covalent Bond Cross-Linked Luminescent Silicone Elastomer with Self-Healing and Recyclable Properties. Macromol. Rapid Commun..

[62] Fawcett A.S., Hughes T.C., Zepeda-Velazquez L., Brook M.A. (2015). Phototunable Cross-Linked Polysiloxanes. Macromolecules.

[63] Zuo Y., Lu H., Xue L., Wang X., Wu L., Feng S. (2014). Polysiloxane-based luminescent elastomers prepared by thiol-ene “click” chemistry. Chem. Eur. J..

[64] Song M., Wang Y., Zhang L., Lu H., Feng S. (2019). A Multifunctional Imidazolium-Based Silicone Material with Conductivity, Self-Healing, Fluorescence, and Stretching Sensitivity. Macromol. Rapid Commun..

[65] Buffa M., Carturan S., Debije M.G., Quaranta A., Maggioni G. (2012). Dye-doped polysiloxane rubbers for luminescent solar concentrator systems. Sol. Energy Mater. Sol. Cells.

[66] Sato K., Fukata N., Hirakuri K., Murakami M., Shimizu T., Yamauchi Y. (2010). Flexible and transparent silicon nanoparticle/polymer composites with stable luminescence. Chem. Asian J..

[67] Hu G., Sun Y., Zhuang J., Zhang X., Zhang H., Zheng M., Xiao Y., Liang Y., Dong H., Hu H. (2020). Enhancement of Fluorescence Emission for Tricolor Quantum Dots Assembled in Polysiloxane toward Solar Spectrum-Simulated White Light-Emitting Devices. Small.

[68] Sun R., Feng S., Wang D., Liu H. (2018). Fluorescence-Tuned Silicone Elastomers for Multicolored Ultraviolet Light-Emitting Diodes: Realizing the Processability of Polyhedral Oligomeric Silsesquioxane-Based Hybrid Porous Polymers. Chem. Mater..

[69] Lin Y., Kouznetsova T.B., Craig S.L. (2020). A Latent Mechanoacid for Time-Stamped Mechanochromism and Chemical Signaling in Polymeric Materials. J. Am. Chem. Soc..

[70] Xie Z., Wang F., Liu C.-Y. (2012). Organic-inorganic hybrid functional carbon dot gel glasses. Adv. Mater..

[71] Sun X., Wang Y., Lei Y. (2015). Fluorescence based explosive detection: From mechanisms to sensory materials. Chem. Soc. Rev..

[72] Sun R., Huo X., Lu H., Feng S., Wang D., Liu H. (2018). Recyclable fluorescent paper sensor for visual detection of nitroaromatic explosives. Sens. Actuators B Chem..

[73] Wan W.-M., Tian D., Jing Y.-N., Zhang X.-Y., Wu W., Ren H., Bao H.-L. (2018). NBN-Doped Conjugated Polycyclic Aromatic Hydrocarbons as an AIEgen Class for Extremely Sensitive Detection of Explosives. Angew Chem. Int. Ed..

[74] Mako T.L., Racicot J.M., Levine M. (2019). Supramolecular Luminescent Sensors. Chem. Rev..

[75] Gou Z., Zhang X., Zuo Y., Tian M., Dong B., Lin W. (2019). Pyrenyl-Functionalized Polysiloxane Based on Synergistic Effect for Highly Selective and Highly Sensitive Detection of 4-Nitrotoluene. ACS Appl. Mater. Interfaces.

[76] Gou Z., Zhang X., Zuo Y., Tian M., Dong B., Tang Y., Lin W. (2021). Triphenylamine-based silsesquioxane derivatives for multiple anion recognition via anion effect and solvent effect. Sens. Actuators B Chem..

[77] Lv Z., Chen Z., Feng S., Wang D., Liu H. (2022). A sulfur-containing fluorescent hybrid porous polymer for selective detection and adsorption of Hg^2+^ ions. Polym. Chem..

[78] Chanmungkalakul S., Ervithayasuporn V., Hanprasit S., Masik M., Prigyai N., Kiatkamjornwong S. (2017). Silsesquioxane cages as fluoride sensors. Chem. Commun..

[79] Liu H., Chen Z., Feng S., Wang D., Liu H. (2019). A Selenone-Functionalized Polyhedral Oligomeric Silsesquioxane for Selective Detection and Adsorption of Hg^2+^ ions in Aqueous Solutions. Polymers.

[80] Pantuso E., De Filpo G., Nicoletta F.P. (2019). Light-Responsive Polymer Membranes. Adv. Opt. Mater..

[81] Gossweiler G.R., Hewage G.B., Soriano G., Wang Q., Welshofer G.W., Zhao X., Craig S.L. (2014). Mechanochemical Activation of Covalent Bonds in Polymers with Full and Repeatable Macroscopic Shape Recovery. ACS Macro Lett..

[82] An Z., Shan T., He H., Ma M., Shi Y., Chen S., Wang X. (2021). Contradiction or Unity? Thermally Stable Fluorescent Probe for In Situ Fast Identification of Self-sort or Co-assembly of Multicomponent Gelators with Sensitive Properties. ACS Appl. Mater. Interfaces.

[83] Heras D., Reig M., Llorca-Isern N., Garcia-Amorós J., Velasco D. (2019). Highly Efficient Elastomeric Fluorescence Sensors for Force Detection. ACS Appl. Polym. Mater..

[84] Zuo Y., Yang T., Zhang Y., Gou Z., Tian M., Kong X., Lin W. (2018). Two-photon fluorescent polysiloxane-based films with thermally responsive self switching properties achieved by a unique reversible spirocyclization mechanism. Chem. Sci..

[85] Zuo Y., Zhang Y., Dong B., Gou Z., Yang T., Lin W. (2019). Binding Reaction Sites to Polysiloxanes: Unique Fluorescent Probe for Reversible Detection of ClO(-)/GSH Pair and the in Situ Imaging in Live Cells and Zebrafish. Anal. Chem..

[86] Zuo Y., Tian M., Sun J., Yang T., Zhang Y., Lin W. (2018). Silica Nanoparticles with Up-conversion Fluorescence Based on Triplet-Triplet Annihilation Mechanism for Specific Recognition of Apoptosis Cells. Anal. Chem..

[87] Zuo Y., Zhang Y., Gou Z., Lin W. (2019). Facile construction of imidazole functionalized polysiloxanes by thiol-ene “Click” reaction for the consecutive detection of Fe^3+^ and amino acids. Sens. Actuat. B Chem..

[88] Sun C.-L., Li J., Wang X.-Z., Shen R., Liu S., Jiang J.-Q., Li T., Song Q.-W., Liao Q., Fu H.-B. (2019). Rational Design of Organic Probes for Turn-On Two-Photon Excited Fluorescence Imaging and Photodynamic Therapy. Chem.

[89] Ye X., Xiang Y., Wang Q., Li Z., Liu Z. (2019). A Red Emissive Two-Photon Fluorescence Probe Based on Carbon Dots for Intracellular pH Detection. Small.

[90] Jin H., Yang M., Sun Z., Gui R. (2021). Ratiometric two-photon fluorescence probes for sensing, imaging and biomedicine applications at living cell and small animal levels. Coord. Chem. Rev..

[91] Chen B., Feng G., He B., Goh C., Xu S., Ramos-Ortiz G., Aparicio-Ixta L., Zhou J., Ng L., Zhao Z. (2016). Silole-Based Red Fluorescent Organic Dots for Bright Two-Photon Fluorescence In vitro Cell and In vivo Blood Vessel Imaging. Small.

[92] Owens E.A., Henary M., El Fakhri G., Choi H.S. (2016). Tissue-Specific Near-Infrared Fluorescence Imaging. Acc. Chem. Res..

[93] Ji Y., Jones C., Baek Y., Park G.K., Kashiwagi S., Choi H.S. (2020). Near-infrared fluorescence imaging in immunotherapy. Adv. Drug Deliv. Rev..

[94] Koide Y., Urano Y., Hanaoka K., Piao W., Kusakabe M., Saito N., Terai T., Okabe T., Nagano T. (2012). Development of NIR Fluorescent Dyes Based on Si–rhodamine for in Vivo Imaging. J. Am. Chem. Soc..

[95] Wirth R., Gao P., Nienhaus G.U., Sunbul M., Jäschke A. (2019). SiRA: A Silicon Rhodamine-Binding Aptamer for Live-Cell Super-Resolution RNA Imaging. J. Am. Chem. Soc..

[96] Zuo Y., Wang X., Gou Z., Lin W. (2020). Step-wise functionalization of polysiloxane towards a versatile dual-response fluorescent probe and elastomer for the detection of H_2_S in two-photon and NO in near-infrared modes. Chem. Commun..

[97] Liu H., Cheng G., Hu D., Shen F., Lv Y., Sun G., Yang B., Lu P., Ma Y. (2012). A Highly Efficient, Blue-Phosphorescent Device Based on a Wide-Bandgap Host/FIrpic: Rational Design of the Carbazole and Phosphine Oxide Moieties on Tetraphenylsilane. Adv. Funct. Mater..

[98] Li Z., Kong J., Wang F., He C. (2017). Polyhedral oligomeric silsesquioxanes (POSSs): An important building block for organic optoelectronic materials. J. Mater. Chem. C.

[99] Yu T., Xu Z., Su W., Zhao Y., Zhang H., Bao Y. (2016). Highly efficient phosphorescent materials based on Ir(iii) complexes-grafted on a polyhedral oligomeric silsesquioxane core. Dalton Trans..

[100] Ertan S., Cihaner A. (2018). Designing a Solution Processable Poly(3,4-ethylenedioxyselenophene) Analogue. Macromolecules.

[101] Cheng C.-C., Chu Y.-L., Chu C.-W., Lee D.-J. (2016). Highly efficient organic–inorganic electroluminescence materials for solution-processed blue organic light-emitting diodes. J. Mater. Chem. C.

[102] Hu G., Xu X., Lei B., Zhuang J., Zhang X., Zhang H., Hu C., Liu X., He Y., Liu Y. (2020). Self-formed C-dot-based 2D polysiloxane with high photoluminescence quantum yield and stability. Nanoscale.

